# Netupitant-palonosetron (NEPA) for Preventing Chemotherapy-induced Nausea and Vomiting: From Clinical Trials to Daily Practice

**DOI:** 10.2174/1568009622666220513094352

**Published:** 2022-09-20

**Authors:** Matti Aapro, Karin Jordan, Florian Scotté, Luigi Celio, Meinolf Karthaus, Eric Roeland

**Affiliations:** ^1^ Genolier Cancer Centre, Clinique de Genolier, Genolier, Switzerland;; ^2^ Department Haematology, Oncology and Palliative Care, Ernst von Bergmann Klinikum Potsdam, Potsdam, Germany;; ^3^ Department of Medicine V, Hematology, Oncology and Rheumatology, University Hospital Heidelberg, Heidelberg, Germany;; ^4^ Interdisciplinary Cancer Course Department, Gustave Roussy Cancer Institute, Villejuif, France;; ^5^ Medical Oncology Unit, ASST del Garda, Desenzano del Garda (BS), Italy;; ^6^ Department of Hematology, Oncology and Palliative Care, Klinikum Neuperlach, Munich, Germany;; ^7^ Department of Hematology, Oncology and Palliative Care, Klinikum Harlaching, Munich, Germany;; ^8^ Oregon Health and Sciences University, Knight Cancer Institute, Portland, OR, USA

**Keywords:** Netupitant-palonosetron (NEPA), chemotherapy-induced nausea and vomiting (CINV), NK_1_ receptor antagonist, 5-HT_3_ receptor antagonist, quality of life, antiemetic regimens

## Abstract

Chemotherapy-induced nausea and vomiting (CINV) is a common adverse event associated with many anticancer therapies and can negatively impact patients' quality of life and potentially limit the effectiveness of chemotherapy. Currently, CINV can be prevented in most patients with guideline-recommended antiemetic regimens. However, clinicians do not always follow guidelines, and patients often face difficulties adhering to their prescribed treatments. Therefore, approaches to increase guideline adherence need to be implemented. NEPA is the first and only fixed combination antiemetic, composed of netupitant (oral)/fosnetupitant (intravenous) and palonosetron, which, together with dexamethasone, constitute a triple antiemetic combination recommended for the prevention of CINV for patients receiving highly emetogenic chemotherapy and for certain patients receiving moderately emetogenic chemotherapy. Thus, NEPA offers a convenient and straightforward antiemetic treatment that could improve adherence to guidelines. This review provides an overview of CINV, evaluates the accumulated evidence of NEPA's antiemetic activity and safety from clinical trials and real-world practice, and examines the preliminary evidence of antiemetic control with NEPA in daily clinical settings beyond those described in pivotal trials. Moreover, we review the utility of NEPA in controlling nausea and preserving patients’ quality of life during chemotherapy, two major concerns in managing patients with cancer.

## 1 INTRODUCTION

### 1.1 Chemotherapy-induced Nausea and Vomiting (CINV)

CINV is a common and distressing side effect associated with specific chemotherapeutic drugs and regimens [1-4] that negatively influences the quality of life (QOL) of patients [5] and can lead to suboptimal completion of cancer treatments [6]. Prevention of CINV is a vital part of patient-centered supportive care. Clinicians should focus on delivering the best possible anticancer treatment and correctly managing the adverse events associated with it and the cancer across the disease continuum [7]. In addition, the appropriate monitoring of adverse events, such as CINV, through patient-reported outcomes programs may ultimately impact patients’ overall survival [8].

CINV is classified into five categories on the basis of the timing of CINV occurrence with respect to chemotherapy administration and previous history of CINV (Table **1** [9-15]). The emetic response triggered by chemotherapeutic agents is initiated by the release of several neurotransmitters, including serotonin, substance P, and dopamine, which specifically activate 5-hydroxytryptamine-3 (5-HT_3_), neurokinin-1 (NK_1_), and dopamine-type 2 receptors, respectively, located in central (central pattern generator) and peripheral nervous systems [1, 16]. Acute emesis (0-24 h after initiation of chemotherapy) is primarily triggered by the release of serotonin by enterochromaffin cells in the gastrointestinal tract, followed by the activation of 5-HT_3_ receptors in the intestine, which transmit the signal to the brainstem to process the emetic reflex; to a lesser extent, substance P also plays a role in acute emesis. Various agents can induce delayed emesis (24-120 h after initiation of chemotherapy), which occurs mainly *via* the release of substance P in the brain, followed by the activation of NK_1_ receptors. Next, the dorsal vagal complex, comprising the vomiting center, area postrema, and vagal afferents, consolidates the stimuli and signals the abdominal muscles, stomach, and diaphragm to induce the emetic response [16]. The pathophysiology of nausea is far less understood [17, 18], and because it is a subjective symptom, its assessment is an ongoing clinical challenge [19]. The antiemetics field has evolved dramatically over the past few years with the development of 5-HT_3_ and NK_1_ receptor antagonists (RAs), typically used for CINV treatment in the acute and delayed phases, respectively.

Individual chemotherapeutic agents and their combinations are ranked into four emetogenic levels according to their potential to induce acute emesis in the absence of effective antiemetic prophylaxis (Table **2**) [9, 14]) [20, 21]. This classification sets the grounds for the antiemetic recommendations issued by the different cancer societies.

### 1.2 Antiemetic Guidelines and Adherence to Guidelines Recommendations

Multiple associations, including the Multinational Association of Supportive Care in Cancer (MASCC)/European Society for Medical Oncology (ESMO) [22, 23], American Society of Clinical Oncology (ASCO) [21], and the National Comprehensive Cancer Network (NCCN) (NCCN guidelines, 2021) [10], have issued evidence-based guidelines for antiemetic control in patients with cancer (Table **3**) [10, 21-23]. In general, the triple combination of an NK_1_ RA, a 5-HT_3_ RA, and a corticosteroid (most often dexamethasone) is recommended for patients receiving highly emetogenic chemotherapy (HEC), including anthracycline-cyclophos-phamide (AC) regimens, carboplatin-based regimens (considered HEC by ASCO and NCCN and moderately emetogenic chemotherapy [MEC] by MASCC/ESMO), and for specific patients receiving MEC. When used per guideline recommendations, currently available antiemetic agents can effectively prevent vomiting in most patients [24-26], while preventing nausea is still a medical challenge [19].

Prevention of CINV is inadequate due, in part, to generally low adherence to guidelines [27] both among oncologists [28, 29] and nurses [30, 31]. Several studies have described the low guideline-concordant prescription of antiemetics in daily clinical practice. A large observational study conducted at 35 sites across Europe enrolled a total of 1089 patients with cancer who received HEC or MEC and antiemetic prophylaxis per investigators’ choice [32]. Only 23% of patients received antiemetic prophylaxis consistent with MASCC/ESMO guideline recommendations during both the acute and delayed phases. The most common deviation from guidelines in the HEC setting was the low use of NK_1_ RAs, with 45% of patients receiving just a 5-HT_3_ RA with or without a corticosteroid on day 1. In the MEC setting, 62% of patients received guideline-consistent antiemetic prophylaxis. Importantly, adherence to antiemetic guidelines correlated with higher complete response (CR) rates in both HEC and MEC settings.

These results align with a study that analyzed a data set of real-world prescribing information in Europe that included data representing 489,049 anticancer treatments requiring NK_1_ RA-based antiemetic prophylaxis per MASCC/ESMO guidelines [29]. NK_1_ RAs were prescribed in fewer than half of the patients receiving cisplatin (45%)- or AC (42%)-based chemotherapy and in as few as 19% of those receiving carboplatin-based regimens; guideline-consistent prophylaxis with NK_1_ RA-5-HT_3_ RA-dexamethasone on day 1 was prescribed only in 18%, 24%, and 7% of chemotherapy treatments, respectively. Importantly, underestimating the emetic risk of chemotherapy was one potential reason for nonadherence. In a similar analysis of a database, including data collected between 2012 and 2018 from 217 physicians in the US, 35% of clinicians prescribing cisplatin (n=2543 courses)- and 58% prescribing AC (n=1490 courses)-based regimens complied with guideline recommendations [33]. Exclusion of the NK_1_ RA from the prophylactic regimen was the main reason for nonadherence. Additional factors contributing to low guideline adherence include mistakes by patients, who often face challenges following antiemetic regimens as prescribed, especially during home administration [28, 34]. More recently, a prospective observational study in Italy (NAVY) assessed the incidence of CINV in 246 patients with breast cancer scheduled for AC-based chemotherapy. Nearly all (99%) patients received a 5-HT_3_ RA with dexamethasone for acute CINV prophylaxis, while the NK_1_ RA aprepitant was used in combination with a 5-HT_3_ RA and dexamethasone, consistent with national guidelines, in less than half (46%) of the patients. Notably, adherence to antiemetic guidelines was associated with a 90% increase in achieving complete protection during the overall period [35].

The complexity of antiemetic regimens may influence adherence to antiemetic guidelines regarding the number of doses and treatment schedules, which is largely determined by choice of NK_1_ RA and the use of three- *vs.* four-drug prophylactic regimens. Simple regimens may improve adherence to guideline recommendations by easing clinicians’ prescription of guideline-consistent antiemetics and increasing patient adherence. Ultimately, this convenience may improve antiemetic control in real-world clinical practice [28, 36, 37].

In this review, we provide an overview of the efficacy and safety of the only fixed combination antiemetic, NEPA, netupitant (oral)/fosnetupitant (intravenous [IV]) with palonosetron in the prevention of CINV. We discuss the results of the pivotal randomized controlled NEPA trials and how these compare with recent outcomes from studies in real-world settings. We examine preliminary evidence of the antiemetic effects of NEPA beyond the settings described in pivotal trials. In addition, we compare prophylactic outcomes between NEPA and aprepitant, the first approved NK_1_ RA. Finally, we analyze the results that support NEPA's use in the control of nausea and preservation of patients’ QOL in the disease continuum, the two areas that are the main clinical challenges in the antiemetics field.

## 2 METHODS

This review article summarizes the key studies in the clinical development of NEPA and reports additional findings from post-approval studies and clinical practice experience. There are only a limited number of studies, and no formal literature search was performed. Literature for the background on CINV and antiemetic prophylaxis was selected on the basis of authors’ records, searches for recent literature with a focus on CINV, and the most recent antiemetic guidelines, at the time of manuscript development, available at the societies’ websites (last accessed October 2021) [10, 21-23].

## 3 NEPA

NEPA is the first and only fixed combination antiemetic, which combines the highly selective NK_1_ RA netupitant (300 mg, for oral use) or fosnetupitant (235 mg, for IV use) with the pharmacologically and clinically distinct second-generation 5-HT_3_ RA palonosetron (0.50 mg, oral; 0.25 mg, IV). Netupitant and palonosetron have complementary pharmacokinetic (PK) profiles, lack PK interactions, and have synergistic inhibitory effects on NK_1_ receptors. Thus, NEPA targets the two main emetic pathways for effective CINV prophylaxis, covering the overall (0-120 h) post-chemotherapy period [38, 39]. NEPA is administered as a single oral dose approximately 60 minutes before chemotherapy or as an IV infusion 30 minutes before the start of chemotherapy. Consequently, NEPA offers the simplicity and convenience of administration to potentially improve guideline-consistent antiemetics prescription by clinicians and treatment adherence by patients. In 2014, the US Food and Drug Administration (FDA) approved oral NEPA for the prevention of acute and delayed CINV associated with single or multiple cycles of HEC and MEC on the basis of the efficacy results from three pivotal clinical trials with oral NEPA-dexamethasone in chemotherapy-naive patients [38, 40-42]. The European Medicines Agency (EMA) approved the use of oral NEPA in 2015 for the prevention of acute and delayed CINV in patients receiving cisplatin-based HEC and MEC [38]. NEPA provided effective antiemetic prophylaxis, including nausea control, in patients with different types of cancer irrespective of sex or age. This approval was followed by immediate uptake by the major cancer societies and NEPA's inclusion in their antiemetic guidelines [10, 21-23]. IV NEPA was approved by the FDA (2018), and the EMA (2019), and antiemetic guidelines recommend its use interchangeably with that of oral NEPA to offer an alternative formulation to improve CINV management [43], providing additional convenience for patients (particularly those with swallowing difficulties) and clinicians.

## 4 NEPA CLINICAL DEVELOPMENT

### 4.1 Pivotal Clinical Trials with NEPA: Efficacy and Safety

NEPA's antiemetic efficacy has been investigated in multiple randomized controlled trials, required by the regulatory authorities for registration, and is summarized in Tables **4** [40-42, 44-51] and **5** [40-42, 44, 46-51]. In the HEC setting, oral NEPA-dexamethasone was superior to palonosetron-dexamethasone in terms of CR and no significant nausea (NSN) in the acute, delayed, and overall phases [42]. In the only head-to-head phase 3 efficacy and safety study comparing two NK_1_ RAs, a single dose of oral NEPA-dexamethasone was non-inferior, in terms of overall CR rate, to the three-day oral aprepitant-granisetron-dexamethasone regimen. Additionally, oral NEPA had a significant benefit compared with the aprepitant regimen regarding the frequency of patients who did not need rescue medication in the delayed and overall phases. While the differences between groups were not statistically significant for no emesis and NSN in the delayed and overall phases, they were numerically higher for patients treated with oral NEPA [44]. Notably, daily rates of breakthrough CINV declined gradually over the 5 days with oral NEPA while remaining constant with the aprepitant regimen, with significantly lower rates observed for oral NEPA on day 5 (8% *vs.* 14%, *p*=0.006) [52]. Regarding the different NEPA formulations, efficacy with IV NEPA was similar to oral NEPA and in line with previous studies in the HEC-cisplatin setting [42, 44].

In patients receiving AC-based regimens, CR rates with oral NEPA-dexamethasone were significantly higher than with palonosetron-dexamethasone in the acute, delayed, and overall periods after a single cycle of chemotherapy and were sustained across multiple cycles. In addition, NSN rates were significantly lower with oral NEPA in the delayed and overall phases [40]. Regarding IV NEPA, the antiemetic activity in cycle 1 was similar to that of the oral formulation for all efficacy endpoints analyzed and was sustained for four cycles [47].

In the non-AC HEC and MEC settings, oral NEPA efficacy has been described in a phase 3 safety study in patients randomized 3:1 to oral NEPA-dexamethasone or aprepitant-palonosetron-dexamethasone [41]. Although efficacy was a secondary objective and no statistical analysis was performed between arms, patients receiving oral NEPA achieved numerically higher CR rates than those receiving the aprepitant regimen in the overall and delayed periods. Similarly, NSN rates were high with oral NEPA and numerically superior to those with aprepitant in the delayed and overall phases [41].

NEPA safety has been well-characterized in multiple pivotal studies in diverse chemotherapy settings. In total,2387 patients treated with the oral and IV formulations in single or repeated cycles have been assessed. NEPA has consistently shown good tolerability with a favorable safety profile (Table **6**) [40-42, 44, 45, 47, 50, 51, 53]) [40-42, 44-47]. Oral NEPA-related adverse events (AEs) were mainly mild or moderate in intensity. The most common were constipation, hiccups, and headaches, in line with those reported for NK_1_ RAs and 5-HT_3_ RAs. In addition, oral NEPA was well tolerated over single and multiple chemotherapy cycles, with no cardiac safety concerns reported [40-42, 44]. The incidence and profile of treatment-emergent AEs (TEAEs) for IV NEPA were similar to those of oral NEPA [45, 47]. Importantly, IV NEPA administration was not associated with treatment-related injection-site, hypersensitivity, or anaphylactic AEs [45, 47].

### 4.2 Post-approval Experience with Oral NEPA: Antiemetic Control and Safety

Randomized controlled trials are the “gold standard” for generating evidence of a drug's efficacy and safety. However, because they are performed under strict conditions that comply with regulatory authorities' requirements, the enrollment criteria, timelines, and atypical comparators, their extrapolation to standard clinical practice can be limited. Real-world data provide information regarding the effectiveness and tolerability of drugs in daily practice and their impact on resource use, medical costs, pharmacoeconomic outcomes, and patient-reported outcomes. Consequently, postmarketing studies are increasingly required. Overall, the studies in everyday clinical practice with oral NEPA administered per label recommendations confirm the antiemetic activity and safety observed in clinical trials.

#### 4.2.1 Real-world Studies of Oral NEPA Use in Approved Clinical Settings

Since its approval, NEPA has been extensively used to prevent CINV in daily clinical practice. This use has facilitated capturing valuable information in various patients with diverse baseline characteristics and clinical settings, reflecting CINV prophylaxis outcomes with NEPA in the real world. Two prospective studies in daily-practice settings where oral NEPA was administered (per label specifications) have been conducted in different countries. A large observational study (AkyPRO) enrolled 2173 patients at 162 centers throughout Germany to investigate the clinical outcomes with up to three cycles of oral NEPA in the HEC and MEC settings [51]. The effectiveness of oral NEPA was similar to that reported in clinical trials for CR, no emesis, and no rescue medication endpoints, while it was slightly lower compared with clinical trials for NSN and no-nausea endpoints (Tables **4** [40-42, 44-51] and **5** [40-42, 44, 46-51]). Conversely, a real-world study with an aprepitant/fosapre-pitant-palonosetron-dexamethasone regimen showed decreased effectiveness under daily clinical practice conditions than the efficacy reported in clinical trials, which may suggest greater difficulty in implementing this regimen in clinical practice [54]. In the AkyPRO study, it was notable that most physicians (≥89%) and patients (≥86%) rated the effectiveness of NEPA prophylaxis as “very good” or “good” during the three chemotherapy cycles, which may reveal a clear benefit of this regimen in the real world [51]. Importantly, the oral NEPA safety profile in daily practice largely mirrored that observed in clinical trials. Investigators completed a subanalysis of the AkyPRO study in 1197 patients with breast cancer receiving AC-based chemotherapy [50]. In cycle 1, the CR and no-emesis rates were consistent during the acute, delayed, and overall periods with clinical trial data in the AC setting, while NSN rates were slightly lower, especially in the acute phase [40].

The second study was a multicenter, observational, real-world study conducted in Canada (EVOLVE; NCT03649230) [46] in 197 patients receiving oral NEPA to prevent CINV associated with HEC, where nearly half of the patients (47%) received AC, 21% cisplatin, and 22% carboplatin (area under the concentration-time curve ≥4 mg/mL/min). While the primary objective was to assess patient-reported QOL with oral NEPA, effectiveness and safety were also evaluated. Oral NEPA was highly effective in CINV control in cycle 1 (Table **4**) [40-42, 44-51], and CR and NSN rates during the acute, delayed, and overall periods increased gradually in cycles 2 through 4, and the need to use rescue medication decreased consistently across cycles. In general, oral NEPA was well-tolerated. Most treatment-related AEs were mild to moderate in severity, the most common being constipation (Table **7**) [41, 45-47, 50, 51, 53].

#### 4.2.2 Interventional Studies of Oral NEPA Use in Approved Clinical Settings

Recently, two prospective, nonrandomized, single-arm studies, which were not part of the clinical development program required by regulatory agencies, assessed oral NEPA in patients with breast cancer treated with AC. The first was a phase 2 study conducted in Italy in 139 patients receiving four cycles of AC-based chemotherapy [48]. With an overall CR rate of 71% in cycle 1, oral NEPA efficacy was comparable to that in the pivotal phase 3 trial (74%) [40]. Remarkably, the overall CR rate was maintained in subsequent cycles, with most patients who achieved CR in cycle 1 sustaining it over cycles 2-4. Generally, oral NEPA treatment was well tolerated without evidence of increased toxicity across cycles. The second study was completed at two centers in China and enrolled a total of 60 patients receiving neo-/adjuvant AC for breast cancer, many of whom presented additional risk factors [49]; 35% had a history of motion sickness, and 40% had experienced vomiting during pregnancy. The CR rates were 70%, 86%, and 60% in the acute, delayed, and overall phases, respectively; the rates of NSN were 87%, 90%, and 78%, and rates of no nausea were 70%, 76%, and 53%, respectively. Further, a previous randomized study compared oral NEPA prophylaxis effects with a historical control of patients who received aprepitant-ondansetron-dexamethasone. Similar CR rates were observed in the acute (70% *vs.* 72%) phase for oral NEPA- and the historical aprepitant-treated groups. In contrast, CR rates in the delayed (86% *vs.* 64%) and overall (60% *vs.* 47%) phases were higher with oral NEPA than with aprepitant. These effects were maintained across four chemotherapy cycles. In addition, oral NEPA treatment showed a benefit over the aprepitant control for delayed and overall NSN (acute: 87% *vs.* 89%; delayed: 90% *vs.* 74%; overall: 78% *vs.* 66%). Moreover, oral NEPA treatment was well tolerated.

#### 4.2.3 Interventional Studies of Oral NEPA Use in Additional Clinical Settings

##### 4.2.3.1 Outcomes with Oral NEPA after 5-HT_3_ RA Failure

Since its approval, the use of NEPA has been explored in other clinical settings beyond those described in pivotal trials to address different current clinical needs. Prophylaxis with oral NEPA-dexamethasone for rescuing patients who experienced CINV despite prior 5-HT_3_ RA-dexamethasone treatment has been analyzed in various single-center studies under real-world conditions. In one study, the use of oral NEPA with dexamethasone was investigated in patients with different solid tumors who had experienced CINV while receiving the first cycle of carboplatin-gemcitabine, despite antiemetic treatment with 5-HT_3_ RA-dexamethasone [55]. Among the 30 patients enrolled, 15 (50%) experienced CINV during cycle 1 and were switched to oral NEPA for subsequent cycles. In total, 13 out of the 15 patients (87%) achieved an overall CR during oral NEPA-dexamethasone treatment, and 13 (87%) and 12 (80%) patients achieved complete control of emesis and nausea during the acute and delayed phases, respectively. Oral NEPA treatment was well-tolerated, with only two patients experiencing grade 1 constipation. Another study retrospectively investigated oral NEPA-dexamethasone in patients with Hodgkin’s lymphoma who had experienced CINV with previous palonosetron-dexamethasone prophylaxis for the doxorubicin, bleomycin, vinblastine, and dacarbazine (ABVD) combination [56]. For 15 out of the 32 patients (47%) treated with ABVD, palonosetron did not control CINV; therefore, these patients were shifted to oral NEPA in the following cycle. Switching to oral NEPA was not associated with an increase in AEs. Despite previous emesis, eight patients (53%) achieved CINV control for all remaining chemotherapy cycles. While these studies involve a low number of patients, the results indicate that oral NEPA may be highly effective in patients for whom previous 5-HT_3_ RA and dexamethasone prophylaxis had failed for different chemotherapy settings.

##### 4.2.3.2 Oral NEPA in Multiple-day Chemotherapy

CINV control is a serious concern for patients receiving multiple-day chemotherapy regimens, especially for patients with hematologic malignancies undergoing hematopoietic cell transplantation (HCT), because they require high-dose conditioning regimens with HEC and MEC agents administered over multiple days. For these patients, antiemetic guidelines recommend an NK_1_ RA, aprepitant, plus 5-HT_3_ RA-dexamethasone [21, 23], or a four-drug regimen with the addition of olanzapine to the combination [21]. However, CINV control remains a clinical challenge for patients receiving intensive conditioning regimens. Only approximately 60% of patients achieved CR in the overall and delayed phases in phase 3 trials with aprepitant/fosaprepitant-based three- or four-drug antiemetic combinations [57, 58].

On the basis of netupitant’s overall exposure predictions from a PK-modeling study supporting the use of netupitant in multiple-day administration schedules [59], three phase 2 trials have shown evidence for using oral NEPA in patients receiving multiple-day high-dose chemotherapy.

A prospective study in 18 patients with sarcoma receiving multiple-day epirubicin-ifosfamide (on days 1-3 of 21-day cycles) assessed a single oral NEPA dose on day 1 plus dexamethasone on days 1-3 for CINV control over 5 days (3 days of chemotherapy plus 2 days after) [60]. Overall CR rates across cycles 1, 2, and 3 were 89%, 89%, and 82%, respectively. CR rates were high in both the acute (100% in cycle 1, 99% in cycle 2, 94% in cycle 3) and delayed phases (89% in cycle 1, 99% in cycle 2, 88% in cycle 3), and no patients needed rescue medication during the 7 days of assessment. These results suggest that a single oral NEPA dose may offer effective prophylaxis for multiple-day chemotherapy.

Another study investigated whether multiple doses of oral NEPA alone effectively prevented CINV in patients with relapsed/refractory non-Hodgkin lymphoma receiving high-dose multiple-day chemotherapy before autologous stem cell transplantation [61]. Patients received oral NEPA every other day starting from the first day of the conditioning regimen. Dexamethasone was omitted because its immunosuppressive effects can increase the risk of serious infections in an already immunocompromised population. Among the 70 patients participating in the study, the CR rate was 87% in the overall (days 1-8), 89% in the acute (days 1-6), and 99% in the delayed (days 7-8) periods. Oral NEPA also controlled nausea effectively, with daily no-nausea rates of 65%. Notably, the every-other-day oral NEPA administration schedule was well-tolerated, with only one AE (constipation) being reported as related to oral NEPA. Of interest, as dexamethasone can contribute to additional immunosuppression in these patients, it was excluded from the prophylactic regimen; thus, oral NEPA alone was very effective in CINV control in this setting. Finally, a multiple-day NEPA regimen was evaluated in 43 patients undergoing HCT who received BEAM (carmustine, etoposide/cytarabine, and melphalan) conditioning on days 1-6 [62, 63]. Oral NEPA was administered on days 1, 3, and 6 of conditioning and dexamethasone on days 1-6. Thirteen out of the 42 patients (31%) who completed the study achieved CR (defined as no emesis, mild to moderate nausea, and no need for rescue medication); vomiting was completely controlled in all (100%) patients in the acute phase (days 1-6) and in 81% during the delayed phase (days 7-11). Twenty-five (60%) patients had a major response (defined as one or two emetic episodes in 1 day only with any level of nausea or no emesis with severe nausea). The most common AEs possibly related to oral NEPA treatment were constipation (56%), diarrhea (42%), and abdominal pain (37%).

Overall, these results suggest that oral NEPA as a single dose or multiple doses may offer effective prophylaxis for multiple-day chemotherapy. The promising outcomes observed with oral NEPA alone suggest that the tolerability in this immunocompromised population could be improved with the omission of dexamethasone. Overall, the existing preliminary clinical experience indicates that oral NEPA regimens may provide flexibility in administration and can be tailored to specific multiple-day chemotherapy settings.

##### 4.2.3.3 Reduction of Dexamethasone Dose in Oral NEPA Regimens

While NEPA is administered in combination with dexamethasone, oral NEPA alone can also prevent CINV, as seen in the Di Renzo study [61]. This prevention is important because, while dexamethasone has an integral role in CINV management, it is associated with side effects, even with short-term use. Events of insomnia, gastrointestinal symptoms, agitation, increased appetite, weight gain, and skin rash were observed in a study where patients received 10 or 20 mg dexamethasone before MEC and at the physician’s discretion to prevent CINV in the delayed period [64]. In addition, dexamethasone-sparing regimens can help with the added burden of multiple antiemetic medications.

Several studies have investigated whether reducing the frequency and total dose of dexamethasone administration may decrease its toxicity without compromising the antiemetic efficacy. A meta-analysis that included seven randomized controlled trials in the HEC and MEC settings and a total of 659 and 649 patients who received 1-day and 3-day dexamethasone, respectively, showed that both dexamethasone regimens had a similar safety profile and achieved comparable CINV control [65]. Another meta-analysis, including only studies in the MEC or AC-based chemotherapy settings with 1970 patients, demonstrated that palonosetron plus 1- *vs.* 3-day dexamethasone regimens provide similar protection against CINV, including against delayed nausea [66]. In line with this, during the COVID-19 pandemic, ESMO has recommended limiting dexamethasone to only day 1 and at a reduced dose for all chemotherapy settings [67]. Dexamethasone-sparing regimens with oral NEPA have been investigated in patients with lung cancer receiving cisplatin-based chemotherapy in a phase 3 noninferiority study [68]. A total of 228 patients received a single oral dose of NEPA and dexamethasone (12 mg on day 1) before chemotherapy and were then randomized (1:1:1) to receive no further dexamethasone, 4 mg dexamethasone daily (on days 2 and 3), or 4 mg twice daily (on days 2-4) per guidelines. Both dexamethasone-sparing arms were non-inferior to the guideline-recommended arm in terms of overall CR rates (76% *vs.* 75%, respectively). There were no clinically significant differences in the severity of dexamethasone-associated AEs between treatment arms. Similarly, in a previous phase 3 study [69] with a dexamethasone-sparing regimen and aprepitant in patients receiving HEC regimens (cisplatin or AC based), CINV prophylaxis with aprepitant/fosaprepitant and palonosetron plus dexamethasone on day 1 only was non-inferior to standard dexamethasone on days 1-3, in terms of CR in the overall phase (47% *vs.* 44%, respectively). By chemotherapy regimen, the overall CR rate achieved with the prophylactic treatment in which dexamethasone was administered on day 1 only was non-inferior to the 3-day dexamethasone treatment for patients receiving AC-based regimens but not for those receiving cisplatin. Therefore, in the HEC setting, dexamethasone use can be spared on days 2 and 3 for patients receiving NK_1_ RA-based prophylaxis (*i.e.*, NK_1_ RA-5-HT_3_ RA-dexamethasone). The study by Celio *et al.* provides the first evidence for prophylaxis with a dexamethasone-sparing regimen in the cisplatin setting. It demonstrates that oral NEPA alone may effectively protect against CINV [68]. A simplified antiemetic regimen of oral NEPA with reduced-dose dexamethasone may be an alternative for frail patients who experience dexamethasone-related AEs, those with certain comorbidities (*e.g.*, diabetes), or for patients prone to nonadherence with follow-up treatments at home (*e.g.*, elderly, patients without family/caregiver support).

### 4.3 Oral NEPA *vs*. Aprepitant Regimens for CINV Prevention

Oral NEPA with dexamethasone has also been compared with other NK_1_ RA-based antiemetic regimens (Table **8**) [37, 41, 42, 44, 70]. In the above-mentioned phase 3 study supporting the use of oral NEPA in the HEC setting, a single dose of oral NEPA was directly compared with a 3-day aprepitant-granisetron regimen in chemotherapy-naive patients receiving cisplatin; patients in both arms also received dexamethasone on days 1-4 [44]. Oral NEPA (N=412) demonstrated non-inferiority to aprepitant-granisetron (N=416) in terms of overall CR rate (74% *vs.* 72%, respectively). Significantly, more patients treated with oral NEPA did not require the use of rescue medication compared with aprepitant-granisetron-treated patients in the delayed (98% *vs.* 95%) and overall phases (97% *vs.* 94%). The daily rates of patients reporting CINV events (defined as experiencing emetic episodes and/or use of rescue medication) remained constant during the 5-day study period in the aprepitant-granisetron group (13%-15%) and decreased gradually for patients treated with oral NEPA (from 16% on day 1 to 8% on day 5); in an exploratory analysis, the difference between groups was statistically significant on day 5 (8% for oral NEPA *vs.* 14% for aprepitant-granisetron; *p*=0.0063). The frequency and severity of AEs were similar between groups.

Pooled data from registration studies of single-dose oral NEPA (N=621) and 3-day aprepitant (N=576) regimens in patients receiving cisplatin-based HEC have been analyzed retrospectively [70]. Both regimens were similar in the acute phase in terms of complete protection, CR, and NSN. In contrast, oral NEPA was significantly more effective for the three endpoints in the delayed phase and NSN in the overall phase. Additionally, the proportion of patients experiencing breakthrough CINV was significantly lower with oral NEPA on days 3-5. A subset of 405 patients treated with oral NEPA and 353 treated with aprepitant received high-dose cisplatin (≥70 mg/m^2^) [71]. Overall, antiemetic efficacy was lower for all endpoints in both groups compared with the overall population, and this decrease was more pronounced for patients who received aprepitant. The benefit of oral NEPA *vs.* aprepitant in the delayed phase was greater in this subset of patients receiving high-dose cisplatin than in the overall cisplatin population. Moreover, complete protection and NSN rates were significantly higher with oral NEPA than with aprepitant in the overall phase.

Recently, single-dose oral NEPA was compared to a 3-day aprepitant regimen, the standard of care in France, in a real-world setting in a pragmatic, randomized, prospective study [37]. Patients treated with AC and various MEC regimens were included, and randomization was stratified by chemotherapy regimen. In contrast to the oral NEPA pivotal trials, where patients receiving AC and MEC were combined per local regulatory authorities' requirements, patients receiving AC and MEC could be evaluated independently in the Zelek *et al.* study. While all (187/187, 100%) patients received oral NEPA, 89% (165/186) received all three doses of aprepitant. In both groups, most patients received the corticosteroid on day 1; however, approximately half of patients took it on days 2 and 3, and a third of patients on day 4. Noninferiority of oral NEPA compared with aprepitant was demonstrated in terms of overall CR rate in the overall population (65% with oral NEPA *vs.* 54% with aprepitant); CR rates in the acute and delayed phases were 7% and 4% higher, respectively, with oral NEPA. Additionally, no emesis, no need to use rescue medication, and NSN rates in all phases were also numerically higher for patients receiving oral NEPA. By chemotherapy setting, CR rates in the acute, delayed, and overall periods were higher for oral NEPA, both for patients treated with AC and MEC regimens; the most notable difference was the 13% higher overall CR rate in the MEC group with oral NEPA. Of interest, 13% of patients in the AC group experienced less significant nausea in the delayed and overall periods with oral NEPA than aprepitant. Finally, the incidence and profile of AEs and treatment-related AEs were similar in both groups.

Overall, these studies indicate that single-dose oral NEPA is non-inferior to a 3-day aprepitant regimen and suggest that it may offer higher daily CINV protection and better control in the delayed phase. There are also indications of better adherence to the oral NEPA than the aprepitant regimen under real-world conditions.

### 4.4 Unmet Medical Needs with NEPA: Nausea Control

While current antiemetics and antiemetic combinations effectively prevent chemotherapy-associated vomiting, nausea control is still a clinical challenge, especially during the delayed period. Oral NEPA-dexamethasone is the only NK_1_ RA-based regimen that has consistently shown a benefit in nausea control compared with 5-HT_3_ RA-dexamethasone in clinical trials (Table **5**) [40-42, 44, 46-51]) [19]. Oral NEPA has shown significant superiority in nausea control during the delayed and overall periods compared with palonosetron in the cisplatin- and AC-based settings [40, 42] and a numeric advantage compared with aprepitant-based regimens in non-AC-based HEC and MEC settings [41, 44]. In the above-mentioned posthoc pooled analysis of phase 3 studies comparing oral NEPA with aprepitant regimens in the cisplatin setting [70], the benefits of oral NEPA were significantly higher for NSN rates in the delayed and overall periods and breakthrough nausea on days 3-5 after chemotherapy [70]. Remarkably, the differences between regimens were more marked in the subgroup of patients receiving high-dose cisplatin (≥70 mg/m^2^; oral NEPA, n=405; aprepitant, n=353) [71]. Additionally, the effects of oral NEPA in nausea control were sustained over multiple cycles. The higher protection against nausea with oral NEPA than with palonosetron was maintained across four cycles of AC, with overall NSN rates ranging between 75%-80% in the oral NEPA arm *vs.* 69%-75% with oral palonosetron. Although exploratory, this difference between treatments was statistically significant in each cycle [72]. Overall NSN rates were also numerically superior with oral NEPA *vs.* the aprepitant regimen for six cycles of non-AC-based HEC or MEC [41, 72]. The IV and oral NEPA formulations have shown similar efficacy in controlling nausea in patients receiving cisplatin, with 10% and 7% of patients, respectively, experiencing nausea of any grade [45], and in the AC setting across four chemotherapy cycles [47].

Studies in daily clinical settings have shown similar effectiveness for oral NEPA in the prevention of nausea compared with that from clinical trials (Table **5**) [40-42, 44, 46-51]) [47, 49]. The difference in nausea control between randomized trials and real-world studies might reflect disparities in patients’ characteristics. In contrast to the populations enrolled in clinical trials, observational studies included patients non-naive to chemotherapy, who could have experienced nausea in previous therapies, and more patients with comorbidities [51]. In the only study directly comparing oral NEPA with aprepitant regimens in a real-world setting, oral NEPA treatment was at least as effective as the 3-day aprepitant regimen regarding NSN rates in the acute, delayed, and overall periods [37]. This suggests that benefits in nausea control observed with oral NEPA *vs.* other NK_1_ RAs are maintained in daily clinical practice.

### 4.5 NEPA Effects on Patients’ QOL

The negative impact of CINV on patients’ QOL may reduce the completion of planned chemotherapy [5, 6]. Therefore, strategies that improve QOL are critical for the optimal management of patients with cancer. QOL endpoints have been evaluated in several NEPA clinical trials (Table **9**) [40, 44, 46, 47, 50, 51]. In the AC setting, a significantly higher proportion of patients reported no impact on daily life activities due to nausea, vomiting, and combined domains with oral NEPA compared with palonosetron [40]. In the cisplatin setting, more patients receiving oral NEPA than the aprepitant-based regimen reported no impact on daily living due to nausea, vomiting, or both in the acute and delayed periods [44], with differences between groups being statistically significant for the nausea domain in the delayed phase. Notably, similar benefits in QOL were reported for IV and oral NEPA formulations by patients with breast cancer during cycles 1 and 2 of AC therapy [47]. Assessment of QOL during oral NEPA treatment was the primary endpoint in the large observational real-world AkyPRO study [51]. During cycle 1, most patients receiving HEC and MEC reported no impact on daily living due to vomiting, approximately half of patients due to nausea, and around two-thirds due to both nausea and vomiting. This impact remained consistent across subsequent cycles for the vomiting domain, whereas it increased slightly for the nausea domain. In the subanalysis of patients with breast cancer, no effect on daily life due to vomiting, nausea, and both nausea and vomiting was reported by 84%, 53%, and 64% of patients, respectively, in cycle 1, and this increased slightly in cycles 2 and 3 [50]. In the Canadian EVOLVE study in patients receiving HEC, 79% and 50% of patients reported no impact on daily life due to vomiting and nausea, respectively, during the first cycle of oral NEPA [46]. Due to both nausea and vomiting, no impact on daily life was reported by 58% of patients in cycle 1 and increased to 66%, 71%, and 77% in cycles 2, 3, and 4, respectively. Overall, these studies show that the beneficial QOL effects with oral NEPA observed in clinical trials are confirmed in real-world practice.

## 5 ORAL NEPA AND GUIDELINE ADHERENCE IN THE REAL WORLD

Adherence to antiemetic guidelines has consistently improved CINV management compared with nonadherence in the HEC, AC, and MEC settings [24, 26]. Factors influencing guideline adherence in daily clinical practice include guideline-consistent prescription by clinicians, patients’ adherence to prescribed regimens, and treatment tolerability [28, 30, 31]. While guideline-consistent prophylaxis is usually not a concern in clinical trials where drug administration is closely supervised, its effect on CINV control becomes apparent in real-world studies. Reducing the complexity of antiemetic treatments in terms of the number of doses and treatment schedules may be critical to ensuring guideline-consistent prescription and treatment uptake. An overview of the administration schedule of NK_1_ RA-based regimens is depicted in Fig. (**1**). Consequently, three-drug regimens with a low number of doses that require minimal administration of follow-up antiemetics at home by patients would be beneficial. The longer half-life of netupitant (96 h) than aprepitant (9-13 h) allows for single-dose oral NEPA administration per chemotherapy cycle to cover the overall emetic risk period. In contrast, aprepitant needs to be administered on 3 consecutive days. The more convenient administration schedule of oral NEPA leads to higher treatment adherence than with aprepitant regimens in patients receiving AC and MEC in daily practice [37]. With oral NEPA, the NK_1_ and 5-HT_3_ RAs are administered together in a single dose; with aprepitant and rolapitant regimens, the 5-HT_3_ RA is administered separately, and the number of 5-HT_3_ RA doses that are required depends on the agent selected. Regarding the number of corticosteroid doses, owing to the inhibitory effects of both netupitant and aprepitant on the cytochrome P450 3A4 enzyme (CYP3A4), the dose of dexamethasone (mainly metabolized by CYP3A4) is reduced when coadministered with these drugs. In daily clinical practice, adherence to the 4-day dexamethasone treatment was only achieved by a third of patients [37]. A dexamethasone-sparing oral NEPA regimen, consisting of single-dose oral NEPA plus a single dexamethasone 12-mg dose on the day of chemotherapy, was as effective as a 4-day dexamethasone regimen [68]. Therefore, a single oral NEPA and dexamethasone dose only on the day of chemotherapy may provide efficient CINV control. The use of this simplified regimen in the HEC setting in clinical practice requires further analysis. Regarding treatment tolerability, the use of fewer drugs twinned with lower doses reduces the chances of drug-drug interactions and treatment-related AEs. Of note, no significant drug-drug interactions have been identified between netupitant and palonosetron, and it has been shown that the two components have complementary PK profiles [73]. Moreover, reducing the dose of dexamethasone leads to better tolerability for frail patients and patients with preexisting conditions who may not be suitable for corticosteroid treatment [64, 67]. In summary, NEPA offers simple, convenient, and flexible administration that may favor adherence to antiemetic guidelines recommendations in real-world settings.

## CONCLUSION

This review provides an overview of NEPA use in clinical practice, discussing its potential benefits in randomized controlled trials and the real-world setting. After approval of a drug, studies performed in a daily-practice setting are increasingly required by regulatory agencies to provide longitudinal information on the comparative effectiveness and tolerability of drugs, including data on their impact on resource use, medical costs, pharmacoeconomic outcomes, and patient-reported outcomes. Moreover, noninterventional studies are of great value to the clinical community. They can provide real-world evidence regarding the effectiveness and safety of antiemetics, as the patients they enroll have diverse baseline characteristics and clinical settings, which are often more representative of the overall patient population. Furthermore, because these studies do not follow the strict drug administration protocols of clinical trials, information regarding treatment adherence by patients can be collected. Real-world data on the current use of oral NEPA and its benefits for antiemetic prophylaxis are critical to inform clinicians, patients, and policymakers in everyday settings beyond the strict indications and administration regimens per product label.

For the indications and the administration schedule specified in the product label, oral NEPA’s impact on safety and QOL in daily clinical practice aligns with data reported in pivotal clinical trials [46, 49, 50, 51], and high adherence to oral NEPA treatment has been reported [37]. The everyday experience of safety and improved QOL highlights that the benefits of oral NEPA may apply to the global population of patients at risk for CINV; good tolerability and adherence to oral NEPA treatment may contribute to these outcomes. Oral NEPA has also been evaluated in settings outside of the label specifications. The oral combination of NEPA and dexamethasone effectively controlled CINV in patients for whom previous treatment with 5HT_3_ RA-dexamethasone prophylaxis for different chemotherapeutic regimens had failed [55, 56]. Furthermore, oral NEPA has been explored in single- and multiple-dose regimens to prevent CINV during conditioning with multiple-day high-dose chemotherapy in preparation for HCT [60-62]. Multiple doses of oral NEPA were well-tolerated and showed high efficacy in this setting. In contrast, single doses of oral NEPA and dexamethasone on the day of chemotherapy showed similar efficacy to the standard 4-day dexamethasone regimen. Although these studies included a limited number of patients, the encouraging results support further investigation regarding the use of NEPA in various daily clinical practice settings. Finally, oral NEPA consistently showed non-inferiority compared with aprepitant in overall CR and higher control for various efficacy endpoints in CINV's delayed and overall phases [37, 44, 70, 71]. Overall, real-world data support the use of oral NEPA in the settings investigated during its clinical development, confirming its effectiveness and safety in everyday clinical practice and its benefits in patients’ QOL and treatment adherence. In addition, results from oral NEPA prophylaxis in other clinical settings are encouraging and may warrant expanded use of NEPA.

## AUTHORS’ CONTRIBUTIONS

All authors have participated in the preparation of the manuscript and have reviewed and approved the final version.

## Figures and Tables

**Fig. (1):**
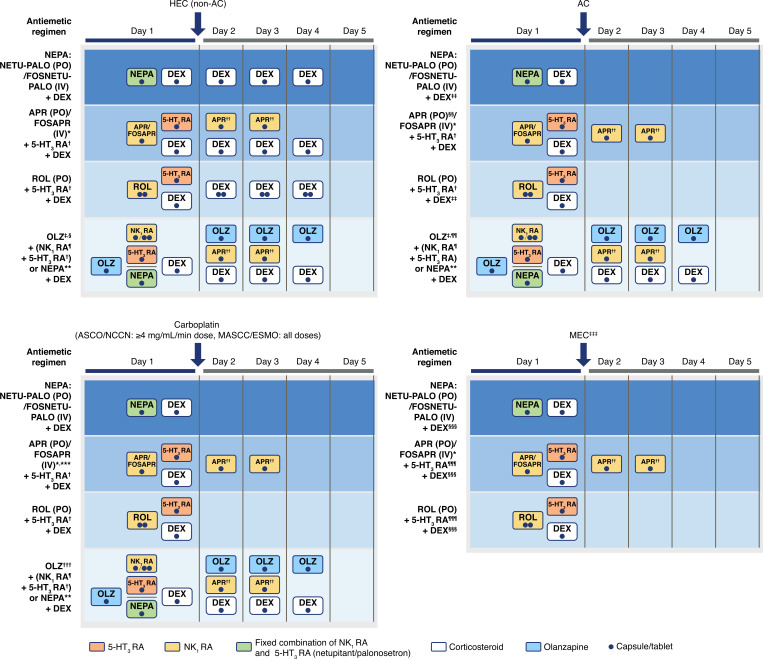
Schedule of oral NK_1_ RA-based antiemetic regimens. **Note:** *NCCN guidelines: aprepitant injectable emulsion IV on day 1 only is also an option. ^†^NCCN, ASCO, and MASCC/ESMO guidelines include dolasetron, granisetron, and ondansetron. In addition, tropisetron is recommended by ASCO and MASCC guidelines and ramosetron by ASCO. ^‡^MASCC guidelines: olanzapine is recommended for patients in whom nausea is a concern. ^§^NCCN guidelines: olanzapine + palonosetron + dexamethasone on day 1 followed by olanzapine on days 2-4 regimen is also an option. ^¶^Includes aprepitant PO, fosaprepitant IV, rolapitant PO. NCCN guidelines also include aprepitant injectable emulsion IV. **Netupitant-palonosetron PO or fosnetupitant-palonosetron IV. ^††^If aprepitant was administered on day 1. ^‡‡^NCCN guidelines: The use of dexamethasone on days 2-4 may be avoided on the basis of patient characteristics; MASCC/ESMO and ASCO guidelines do not recommend the use of dexamethasone on days 2-4. ^§§^MASCC guidelines for the delayed phase: no antiemetic or if aprepitant was administered on day 1, dexamethasone (days 2-4) or aprepitant (days 2, 3) to be administered; olanzapine (days 2-4) to be administered if nausea is a concern. ^¶¶^NCCN guidelines: olanzapine + palonosetron + dexamethasone on day 1 followed by olanzapine on days 2-4 regimen is also an option. ***ASCO guidelines: if fosaprepitant is used on day 1, 100 mg IV to be administered and then 80 mg oral aprepitant on days 2 and 3. ^†††^Olanzapine-based regimens are only recommended in NCCN guidelines: olanzapine + palonosetron + dexamethasone on day 1 followed by olanzapine on days 2-4 regimen is also an option. ^‡‡‡^Regimens recommended in NCCN guidelines for patients with additional risk factors or for whom previous treatment has failed with 5-HT_3_ RA + dexamethasone. ^§§§^Administration of dexamethasone on days 2-3 is an option. ^¶¶¶^NCCN guidelines include dolasetron, granisetron, and ondansetron. **Abbreviations:** 5-HT_3_ RA: 5-hydroxytryptamine-3 receptor antagonist, AC: anthracycline-cyclophosphamide, APR: aprepitant, ASCO: American Society of Clinical Oncology, DEX: dexamethasone, FOSAPR: fosaprepitant, FOSNETU: fosnetupitant, HEC: highly emetogenic chemotherapy, IV: intravenous, MASCC/ESMO: Multinational Association of Supportive Care in Cancer/European Society for Medical Oncology, MEC: moderately emetogenic chemotherapy, NCCN: National Comprehensive Cancer Network, NEPA: netupitant-palonosetron, NETU: netupitant, NK_1_ RA: neurokinin-1 receptor antagonist, OLZ: olanzapine, PALO: palonosetron, PO: oral, ROL: rolapitant.

**Table 1:** Classification of chemotherapy-induced nausea and vomiting.

Classification*	**Definition**
**Acute**	Occurring within the first 24 h after administration of chemotherapy. Often, the intensity peaks after 5-6 h.
**Delayed**	Occurring more than 24 h after administration of chemotherapy and generally lasting until day 5 (days 2-5).
**Breakthrough**	Occurring despite appropriate antiemetic prophylaxis, which often requires rescue medication.
**Anticipatory**	Occurring before chemotherapy administration as a conditioned response to having experienced CINV in previous cycles.
**Refractory**	Recurring in subsequent cycles of therapy when appropriate prophylaxis has failed in previous cycles, excluding anticipatory CINV.

**Note:** *Unrelated to chemotherapy, chronic nausea and vomiting frequently occur in patients with advanced cancer (Navari *et al.* [15]).

CINV: chemotherapy-induced nausea and vomiting

**Table 2:** Classification of emetic risk of chemotherapeutic agents.

**Emetic Risk**	Percentage of Patients with Emesis*
**Minimal**	<10%
**Low**	10%-30%
**Moderate**	30%-90%
**High**	>90%

**Note:** *Percentage of patients with emesis in the absence of effective antiemetic prophylaxis.

**Table 3:** Guideline recommendations for antiemetic use with highly emetogenic, anthracycline-cyclophosphamide-based, carboplatin-based, and moderately emetogenic chemotherapy.

**Emetic Risk Group**	**Highly Emetogenic Chemotherapy**		**Anthracycline-cyclophosphamide Chemotherapy**		**Carboplatin-based ** Chemotherapy**		**Moderately Emetogenic ** **Chemotherapy**	
**Emetic Period**	**Acute ** **Prevention** **Day 1**	**Delayed ** **Prevention** **Days 2-4**	**Acute ** **Prevention** **Day 1**	**Delayed ** **Prevention** **Days 2-4**	**Acute ** **Prevention** **Day 1**	**Delayed ** **Prevention** **Days 2-4**	**Acute ** **Prevention** **Day 1**	**Delayed ** **Prevention** **Days 2-3**
**ASCO [** **21** **]**	NEPA + DEX + OLZ	DEX + OLZ	NEPA + DEX + OLZ	OLZ	NEPA + DEX	None	5-HT_3_ RA + DEX	DEX^¶¶^
	NK_1_ RA (APR/FOS/ROL) + 5-HT_3_ RA + DEX + OLZ	APR^†^ + DEX + OLZ	NK_1_ RA (APR/FOS/ROL) + 5-HT_3_ RA + DEX + OLZ^¶^	APR^†^ + OLZ	NK_1_ RA (APR/FOS/ROL) + 5-HT_3_ RA + DEX	APR^†,††^	-	-
**MASCC/ESMO [** **22** **, ** **23** **]**	NEPA + DEX ± OLZ*	DEX ± OLZ*	NEPA + DEX ± OLZ*	None^§^ ± OLZ*	NEPA + DEX	None	5-HT_3_ RA + DEX	DEX^¶¶^
	NK_1_ RA (APR/FOS/ROL) + 5-HT_3_ RA + DEX ± OLZ*	APR^‡^ + DEX ± OLZ*	NK_1_ RA (APR/FOS/ROL) + 5-HT_3_ RA + DEX ± OLZ*	APR or DEX^¶^ ± OLZ*	NK_1_ RA (APR/FOS/ROL) + 5-HT_3_ RA + DEX	APR^†^	-	-
**NCCN [** **10** **]**	NEPA + DEX ± OLZ	DEX ± OLZ	NEPA + DEX ± OLZ	DEX ± OLZ	NEPA + DEX ± OLZ	DEX ± OLZ	NEPA + DEX^‡‡^	± DEX
	NK_1_ RA (APR/FOS/ROL) + 5-HT_3_ RA + DEX ± OLZ	APR^†^ + DEX ± OLZ	NK_1_ RA (APR/FOS/ROL) + 5-HT_3_ RA + DEX ± OLZ	APR^†^ + DEX ± OLZ	NK_1_ RA (APR/FOS/ROL) + 5-HT_3_ RA + DEX ± OLZ	APR^†^ + DEX ± OLZ	NK_1_ RA (APR/FOS/ROL) + 5-HT_3_ RA + DEX^‡‡^	APR^†^ ± DEX
	-	-	-	-	-	-	5-HT_3_ RA + DEX	5-HT_3_ RA^§§^ or DEX
	PALO + DEX + OLZ	OLZ	PALO + DEX + OLZ	OLZ	PALO + DEX + OLZ	OLZ	PALO + DEX + OLZ^‡‡^	OLZ

**Note:** *Olanzapine may be added, particularly if nausea is a concern.

^†^If aprepitant 125 mg is used on day 1, aprepitant 80 mg on days 2 to 3 is recommended.

^‡^If aprepitant 125 mg is used on day 1, then dexamethasone 8 mg × 1 (days 2-4) + aprepitant 80 mg × 1 (days 2-3) OR dexamethasone 8 mg × 2 (days 2-4) + metoclopramide 20 mg × 4 (days 2-4).

^¶^In non-breast cancer populations (*e.g.,* non-Hodgkin lymphoma) receiving a combination of anthracycline and cyclophosphamide with treatment regimens incorporating corticosteroids, the addition of palonosetron without the use of an NK_1_ RA and olanzapine is an option.

^§^If fosaprepitant, NEPA, or rolapitant has been used on day 1.

^¶^If aprepitant 125 mg is used on day 1, then aprepitant 80 mg × 1 (days 2-3) OR dexamethasone 4 mg × 2 (days 2-3).

**As per ASCO and NCCN guidelines for carboplatin ≥4 mg/mL/min dose; as per MASCC/ESMO guidelines for all carboplatin doses.

^††^If IV aprepitant is used, 100 mg IV on day 1 and then 80 mg oral on days 2-3.

^‡‡^For patients with additional risk factors or for whom previous treatment with a 5-HT_3_ RA + dexamethasone has failed.

^¶¶^For moderate-emetic-risk agents with a known risk for delayed nausea and vomiting (*e.g.*, oxaliplatin, anthracycline, cyclophosphamide).

^§§^No further 5-HT_3_ therapy is required if palonosetron or granisetron extended-release injection is administered, or if a granisetron transdermal patch is applied on day 1.

**Abbreviations:** 5-HT_3_ RA: 5-hydroxytryptamine-3 receptor antagonist, APR: aprepitant, ASCO: American Society of Clinical Oncology, DEX: dexamethasone, ESMO: European Society for Medical Oncology, FOS: fosaprepitant, MASCC: Multinational Association of Supportive Care in Cancer, NCCN: National Comprehensive Cancer Network, NEPA: netupitant-palonosetron, NK_1_ RA: neurokinin-1 receptor antagonist, OLZ: olanzapine, PALO: palonosetron, ROL: rolapitant.

**Table 4:** Summary of CR, no emesis, and no need to use rescue medication with NEPA prophylaxis in clinical trials and real-world studies - a single cycle of chemotherapy.

**Clinical Setting** **Study, Treatment (n)**		**Complete Response, Patients, %**			**No Emesis, Patients, %**			**No Rescue Medication, ** **Patients, %**		
		**Acute**	**Delayed**	**Overall**	**Acute**	**Delayed**	**Overall**	**Acute**	**Delayed**	**Overall**
**Cisplatin-based HEC**										
ClinicalTrial	Hesketh 2014* [**42****]** Oral NEPA_300_ + DEX (n=135) PALO + DEX (n=136)	98.5^†^ 89.7	90.4^‡^ 80.1	89.6^†^ 76.5	98.5^†^ 89.7	91.9^†^ 80.1	91.1^†^ 76.5	100 97.8	98.5 97.1	98.5 95.6
	**Zhang 2018 [****44****]** Oral NEPA + DEX (n=412) APR + GRAN + DEX (n=416)	84.5 87.0	77.9 74.3	73.8 72.4	85.2 87.5	79.4 76.2	75.0 74.0	98.8 98.3	97.6^††^ 94.7	96.6^††^ 93.5
	**Schwartzberg 2018 [****45****]** Oral NEPA + DEX (n=201) IV NEPA + DEX (n=203)	- -	- -	84.1 76.8	- -	- -	88.6 84.2	- -	- -	- -
Real World	**Conter 2020 [****46****]** Oral NEPA + DEX (n=196)	67.8	51.7	46.7	-	-	-	-	-	52.2
**Non-AC-based HEC/MEC**										
Clinical Trial	**Gralla 2014^‡‡^ [****41****]** Oral NEPA + DEX (n=309) APR + PALO + DEX (n=103)	92.9 94.2	83.2 77.7	80.6 75.7	- -	- -	- -	- -	- -	87.7 91.3
**AC-based HEC**										
Clinical Trial	**Aapro 2014 [****40****]** Oral NEPA + DEX (n=724) PALO + DEX (n=725)	88.4^§^ 85.0	76.9^¶^ 69.5	74.3^¶^ 66.6	90.9^§^ 87.3	81.8** 75.6	79.8^¶^ 72.1	93.5 92.3	85.8** 80.6	84.0^§^ 79.0
	**Schwartzberg 2020 [****47****]** Oral NEPA + DEX (n=202) IV NEPA + DEX (n=200)	- -	- -	77.2 73.0	- -	- -	86.1 82.5	- -	- -	86.6 81.5
	**Caputo 2020 [****48****]** Oral NEPA + DEX (n=202)	85.6	72.7	70.5	-	-	-	-	-	-
	**Yeo 2020 [****49****]** Oral NEPA + DEX (n=60)	70.0	85.7	60.0	71.7	86.0	61.7	85.0	90.2	76.7
Real World	**Schilling 2021 [****50****]** Oral NEPA + DEX (n=1197)	86.0	88.2	81.0	94.2	97.1	92.8	90.2	90.1	85.6
**HEC (including AC)/MEC**										
Real World	**Karthaus 2020 [****51****]** Oral NEPA + DEX (n=2153)	89.2	87.1	82.5	95.7	96.1	93.5	92.5	90.0	87.0

**Note:** *Only the results of the NEPA_300_ + DEX arm are shown (commercially approved dose).

^†^ *p*≤0.01 from logistic regression versus PALO + DEX.

^‡^ *p*≤0.05 from logistic regression versus PALO + DEX.

^§^ *p*≤0.05 versus PALO + DEX using a two-sided Cochran-Mantel-Haenszel test stratified by treatment, age group, and region.

^¶^ *p*≤0.001 versus PALO + DEX using a two-sided Cochran-Mantel-Haenszel test stratified by treatment, age group, and region.

**p≤0.01 versus PALO + DEX using a two-sided Cochran-Mantel-Haenszel test stratified by treatment, age group, and region.

^††^ *p*<0.05 versus APR + GRAN + DEX on the basis of the Cochran-Mantel-Haenszel test with gender as a stratifying variable.

^‡‡^Pivotal safety phase 3 study; efficacy outcomes were analyzed descriptively.

**Abbreviations:** AC: anthracycline and cyclophosphamide, APR: aprepitant, CR: complete response, DEX: dexamethasone, GRAN: granisetron, HEC: highly emetogenic chemotherapy, IV: intravenous, MEC: moderately emetogenic chemotherapy, NEPA: netupitant-palonosetron, PALO: palonosetron

**Table 5:** Summary of nausea control with NEPA prophylaxis in clinical trials and real-world studies - the single cycle of chemotherapy.

**Study, Treatment (n)**		**No Significant Nausea, Patients, %**			**No Nausea, Patients, %**		
		**Acute**	**Delayed**	**Overall**	**Acute**	**Delayed**	**Overall**
**Cisplatin-based HEC**							
Clinical Trial	Hesketh 2014* [**42****]** Oral NEPA_300_ + DEX (n=135) PALO + DEX (n=136)	98.5^‡^ 93.4	90.4^†^ 80.9	89.6^‡^ 79.4	- -	- -	- -
	**Zhang 2018 [****44****]** Oral NEPA + DEX (n=412) APR + GRAN + DEX (n=416)	89.8 87.3	78.2 72.8	75.7 70.4	68.9 67.8	53.2 54.3	49.3 51.4
Real World	**Conter 2020^¶^ [****46****]** Oral NEPA + DEX (n=196)	76.2	69.6	64.6	-	-	-
**Non-AC-based HEC/MEC**							
Clinical Trial	**Gralla 2014^§^ [****41****]** Oral NEPA + DEX (n=309) APR + PALO + DEX (n=103)	90.6 93.2	85.1 81.6	84.1 80.6	- -	- -	- -
**AC-based HEC**							
Clinical Trial	**Aapro 2014 [****40****]** Oral NEPA + DEX (n=724) PALO + DEX (n= 25)	87.3 87.9	76.9 71.3	74.6 69.1	- -	- -	- -
	**Schwartzberg 2020 [****47****]** Oral NEPA + DEX (n=202) IV NEPA + DEX (n=200)	85.1 84.0	77.2 72.5	74.8 70.0	- -	- -	48.0 42.0
	**Caputo 2020 [****48****]** Oral NEPA + DEX (n=202)	81.3	61.2	57.6	-	-	-
	**Yeo 2020 [****49****]** Oral NEPA + DEX (n=60)	86.7	90.4	78.3	70.0	76.2	53.3
Real World	**Schilling 2021 [****50****]** Oral NEPA + DEX (n=1197)	69.1	72.7	60.1	43.3	39.7	31.1
**HEC (including AC)/MEC**							
Real World	**Karthaus 2020 [****51****]** Oral NEPA + DEX (n=2153)	78.8	74.8	66.9	55.7	44.3	38.4

**Note:** *Only the results of the NEPA_300_ + DEX arm are shown (commercially approved dose).

^†^ *p*≤0.01 from logistic regression versus PALO + DEX.

^‡^ *p*≤0.05 from logistic regression versus PALO + DEX.

^§^Pivotal safety phase 3 study; efficacy outcomes were analyzed descriptively.

^¶^HEC other than cisplatin included.

**Abbreviations:** AC: anthracycline and cyclophosphamide, AE: adverse event, APR: aprepitant, DEX: dexamethasone, GRAN: granisetron, HEC: highly emetogenic chemotherapy, IV: intravenous, MEC: moderately emetogenic chemotherapy, NEPA: netupitant-palonosetron, PALO: palonosetron, TEAE: treatment-emergent adverse event, TRAE: treatment-related adverse event

**Table 6:** Summary of NEPA safety in pivotal trials and real-world studies following a single cycle of chemotherapy - safety population.

**AE, n (%)**	**Clinical Trials**								**Real-world Studies**	
	**Hesketh 2014^‡^ [** **42** **]**	**Zhang 2018 [** **44** **]**	**Schwartzberg 2018 [** **45** **]**		**Gralla 2014 [** **41** **]**	**Aapro 2014 [** **40** **]**	**Schwartzberg 2020 [** **47** **]**		**Schilling 2021 [** **50** **]**	**Karthaus 2020 [** **51** **]**
	**Cisplatin-based HEC**	**Cisplatin-based HEC**	**Cisplatin-based HEC**		**Non-AC-Based HEC/MEC**	**AC-based HEC**	**AC-based HEC**		**AC-based HEC**	**HEC (incl. AC)/MEC**
	**Oral NEPA_300_ + DEX** **(n=135)**	**Oral NEPA + DEX** **(n=413)**	**Oral NEPA + DEX** **(n=201)**	**IV NEPA + DEX** **(n=203)**	**Oral NEPA + DEX** **(n=308)**	**Oral NEPA + DEX** **(n=725)**	**Oral NEPA + DEX** **(n=202)**	**IV NEPA + DEX** **(n=200)**	**Oral NEPA + DEX** **(n=1192)**	**Oral NEPA + DEX** **(n=2153)**
**Any TEAE**	68 (50.0)	240 (58.1)	135 (67.2)	120 (59.1)	199 (64.6)	551 (76.0)	122 (60.4)	121 (60.5)	286 (24.0)	466 (21.5)
**Serious AE**	0	20 (4.8)	21 (10.4)	29 (14.3)	18 (5.8)	13 (1.8)	1 (0.5)	2 (1.0)	3 (0.3)	88 (4.1)
Any TRAE*	21 (15.4)	76 (18.4)	19 (9.5)	18 (8.9)	16 (5.2)	59 (8.1)	12 (5.9)	13 (6.5)	81 (6.8)	109 (5.0)
Severe TRAE*	0	NS	2 (1.0)	1 (0.5)	1 (0.3)	5 (0.7)	0	0	NS	NS
Serious TRAE*	0	2 (0.5)	0	0	1 (0.3)	0	0	0	3 (0.3)	6 (0.3)
**Most common TRAE^†^ (≥2%)** Constipation Headache Hiccups Increased ALT Fatigue Insomnia Dizziness	- - 7 (5.1) - - - -	33 (8.0) - 11 (2.7) - - - -	NS	NS	7 (2.3) - - - - - -	15 (2.1) 24 (3.3) - - - - -	NS	NS	33 (2.8) - - - 38 (3.2) 25 (2.1) -	- - - - 43 (2.0) - -
TRAE* **leading to ** **discontinuation**	0	1 (0.2)	0	1 (0.5)	1 (0.3)	0	0	0	NS	NS
**Total deaths**	0	0	6 (3.0)^§^	7 (3.4)^§^	7 (2.3)^§^	0	0	0	0	0

**Note:** *Considered by the investigator to be possibly, probably, or definitely related to the study drug (from [53]).

^†^By Medical Dictionary for Regulatory Activities (MedDRA), version 14.0, preferred term.

^‡^Only the results of the NEPA_300_ + DEX arm are shown (commercially approved dose).

^§^All events were judged unrelated to the study drug.

AC: anthracycline and cyclophosphamide, AE: adverse event, ALT: alanine aminotransferase, APR: aprepitant, DEX: dexamethasone, HEC: highly emetogenic chemotherapy, IV: intravenous, MEC: moderately emetogenic chemotherapy, NEPA: netupitant/palonosetron, NS: not specified, TEAE: treatment-emergent adverse event, TRAE: treatment-related adverse event

**Table 7:** Summary of NEPA safety in pivotal trials and real-world studies following repeated cycles of chemotherapy - safety population.

**AE, n (%)**	**Clinical Trials**					**Real-world Studies**		
	**Schwartzberg 2018 ** **[** **45** **]**		**Gralla 2014 [** **41** **]**	**Schwartzberg 2020 ** **[** **47** **]**		**Conter 2020 [** **46** **]**	**Schilling 2021 [** **50** **]**	**Karthaus 2020 [** **51** **]**
	**Cisplatin-based HEC**		**Non-AC-based HEC/MEC**	**AC-based HEC**		**HEC**	**AC-based**	**HEC (incl. AC)/MEC**
	**Oral NEPA + DEX** **(n=201)**	**IV NEPA + ** **DEX** **(n=203)**	**Oral NEPA + DEX** **(n=308)**	**Oral NEPA + DEX** **(n=203)**	**IV NEPA + ** **DEX** **(n=200)**	**Oral NEPA + DEX** **(n=197)**	**Oral NEPA + DEX** **(n=1197)**	**Oral NEPA + DEX** **(n=2173)**
**Any TEAE**	174 (86.6)	169 (83.3)	265 (86.0)	187 (92.1)	184 (92.0)	178 (90.4)	386 (32.2)	650 (29.9)
**Serious AE**	43 (21.4)	41 (20.2)	50 (16.2)	4 (2.0)	5 (2.5)	NS	71 (5.9)	153 (7.0)
Any TRAE*	23 (11.4)	26 (12.8)	31 (10.1)	22 (10.8)	16 (8.0)	49 (24.9)	116 (9.7)	158 (7.3)
Severe TRAE*	3 (1.5)	2 (1.0)	1 (0.3)	1 (0.5)	1 (0.5)	NS	NS	NS
Serious TRAE*	0	0	2 (0.6)	1 (0.5)	0	NS	5 (0.4)	10 (0.5)
**Most common TRAE^†^ (≥2%)** Constipation Headache Hiccups Increased ALT Fatigue Insomnia Nausea Dizziness Abdominal pain	12 (6.0) - - 4 (2.0) - - - - -	13 (6.4) - - 4 (2.0) - - - - -	11 (3.6) - - - - - - - -	5 (2.5) 7 (3.4) - - 4 (2.0) - - 5 (2.5) -	5 (2.5) 5 (2.5) - - 4 (2.0) - - 5 (2.5) -	43 (21.8) 10 (5.1) - - - - - - 4 (2.0)	52 (4.3) 17 (1.4) - - 54 (4.5) 35 (2.9) 25 (2.1) - -	62 (2.9) - - - 64 (3.0) - - - -
TRAE* **leading to discontinuation**	1 (0.5)	2 (1.0)	1 (0.3)	NS	0	NS	NS	NS
**Total deaths**	14 (7.0)^‡^	10 (4.9) ^‡^	16 (5.2)^‡^	0	0	9 (4.6)^‡^	0	0

**Note:** *Considered by the investigator to be possibly, probably, or definitely related to the study drug (from [53]).

^†^By Medical Dictionary for Regulatory Activities (MedDRA), version 14.0, preferred term.

^‡^All events were judged unrelated to the study drug.

**Abbreviations:** AC: anthracycline and cyclophosphamide, AE: adverse event, ALT: alanine aminotransferase, DEX: dexamethasone, HEC: highly emetogenic chemotherapy, IV: intravenous, MEC: moderately emetogenic chemotherapy, NEPA: netupitant-palonosetron, NS: not specified, TEAE: treatment-emergent adverse event, TRAE: treatment-related adverse event

**Table 8:** Summary of antiemetic activity with oral NEPA and aprepitant regimens.

**Outcome, %**	**Clinical Trials**								**Real-world Study**	
	**Zhang 2018 [** **44** **]**		Hesketh 2014* [ **42** **]**		**Gralla 2014 [** **41** **]**		**Navari 2020 [** **70** **]**		**Zelek 2021 [** **37** **]**	
	**Cisplatin-based HEC**		**Cisplatin-based HEC**		**Non-AC-based HEC/MEC**		**Cisplatin-based HEC**		**AC/Non-AC MEC^†^**	
	**Oral NEPA + DEX** **(n=412)**	**APR + GRAN + DEX** **(n=416)**	**Oral NEPA_300_ + DEX** **(n=135)**	**APR + OND + DEX** **(n=134)**	**Oral NEPA + DEX** **(n=309)**	**APR + PALO + DEX** **(n=103)**	**Oral NEPA + DEX** **(n=621)**	**APR + 5-HT_3_ RA + DEX** **(n=576)**	**Oral NEPA + DEX** **(n=188)**	**APR + 5-HT_3_ RA + DEX** **(n=185)**
**Complete response** Acute Delayed Overall	84.5 77.9 73.8	87.0 74.3 72.4	98.5 90.4 89.6	94.8 88.8 86.6	92.9 83.2 80.6	94.2 77.7 75.7	88.4 81.8^§^ 78.4	89.2 76.9 75.0	74.5 90.4 64.9	68.1 85.9 54.1
**No emesis** Acute Delayed Overall	85.2 79.4 75.0	87.5 76.2 74.0	98.5 91.9 91.1	94.8 89.6 87.3	- - -	- - -	- - -	- - -	81.3 90.4 71.7	78.0 88.2 66.1
**No rescue medication** Acute Delayed Overall	98.8 97.6^‡^ 96.6^‡^	98.3 94.7 93.5	- - -	- - -	- - 87.7	- - 91.3	- - -	- - -	85.6 91.4 77.0	82.3 87.1 69.4
**No significant nausea** Acute Delayed Overall	89.8 78.2 75.7	87.3 72.8 70.4	98.5 90.4 89.6	94.0 88.1 85.8	90.6 85.1 84.1	93.2 81.6 80.6	91.9 81.5^§^ 79.5^§^	88.9 76.4 74.1	77.2 70.9 64.8	74.3 63.9 56.4
**No nausea** Acute Delayed Overall	68.9 53.2 49.3	67.8 54.3 51.4	- - -	- - -	- - -	- - -	- - -	- - -	- - -	- - -

**Note:** *Only the results of the NEPA_300_ + DEX arm are shown (commercially approved dose).

^†^For the secondary endpoints (no emesis, no rescue medication, no significant nausea), 187 patients were analyzed in the oral NEPA group and 186 in the APR group.

^‡^ *p* <0.05 versus APR + GRAN + DEX on the basis of the Cochran-Mantel-Haenszel test with gender as a stratifying variable.

^§^ *p* <0.05.

**Abbreviations:** 5-HT_3_ RA: 5-hydroxytryptamine-3, AC: anthracycline and cyclophosphamide, APR: aprepitant, DEX: dexamethasone, GRAN: granisetron, HEC: highly emetogenic chemotherapy, MEC: moderately emetogenic chemotherapy, NEPA: netupitant-palonosetron, OND: ondansetron, PALO: palonosetron.

**Table 9:** Quality of life following antiemetic prophylaxis with NEPA.

**No impact on daily life, %**	**Clinical Trials**						**Real-World Studies**			
	**Zhang 2018 [** **44** **]**		**Aapro 2014 [** **40** **]**		**Schwartzberg 2020 [** **47** **]**		**Conter 2020 [** **46** **]**	**Schilling 2021 [** **50** **]**	**Karthaus 2020 [** **51** **]**	
	**Cisplatin-based HEC**		**AC-based HEC**		**AC-based HEC**		**HEC**	**AC-based**	**HEC (incl. AC)**	**MEC**
	**Oral NEPA + DEX** **(n=412)**	**APR/GRAN + DEX** **(n=416)**	**Oral NEPA + DEX** **(n=724)**	**PALO + DEX** **(n=725)**	**Oral NEPA + DEX** **(n=202)**	**IV NEPA + DEX** **(n=200)**	**Oral NEPA + DEX** **(n=197)**	**Oral NEPA + DEX** **(n=1027)**	**Oral NEPA + DEX** **(n=1198)**	**Oral NEPA + DEX** **(n=688)**
**Nausea domain** Acute Delayed Overall	81.8 71.1^§^ -	80.0 65.1 -	- - 71.5*	- - 65.8	- - 68.3	- - 67.5	- - 49.7	- - 53.0	- - 54	- - 59
**Vomiting domain** Acute Delayed Overall	87.9 81.3 -	86.8 77.4 -	- - 90.1^†^	- - 84.4	- - 90.6	- - 87.5	- - 78.8	- - 84.4	- - 84	- - 82
**Combined domain** Acute Delayed Overall	86.2 76.0 -	83.2 70.7 -	- - 78.5^‡^	- - 72.1	- - 78.7	- - 74.0	- - 58.2	- - 63.8	- - 64	- - 67

**Note:** *p ≤0.05 *versus* PALO + DEX using a two-sided Cochran-Mantel-Haenszel test stratified by treatment, age group, and region.

^†^ *p* ≤0.001 *versus* PALO + DEX using a two-sided Cochran-Mantel-Haenszel test stratified by treatment, age group, and region.

^‡^ *p* ≤0.01 *versus* PALO + DEX using a two-sided Cochran-Mantel-Haenszel test stratified by treatment, age group, and region.

^§^Statistically significant difference versus APR + GRAN + DEX using a two-sided Cochran-Mantel-Haenszel test stratified by gender.

**Abbreviations:** AC: anthracycline and cyclophosphamide, APR: aprepitant, DEX: dexamethasone, GRAN: granisetron, HEC: highly emetogenic chemotherapy, IV: intravenous, MEC: moderately emetogenic chemotherapy, NEPA: netupitant-palonosetron, PALO: palonosetron
